# HEALTH EXP ERIENCES OF SEXUAL AND GENDER MINORITY PEOPLE LIVING WITH DEMENTIA AND THEIR CAREGIVERS: A SCOPING REVIEW

**DOI:** 10.1093/geroni/igac059.2806

**Published:** 2022-12-20

**Authors:** Melissa Harris, Jennifer May

**Affiliations:** Duke University, Durham, North Carolina, United States; Duke, Durham, North Carolina, United States

## Abstract

People living with dementia and older adults who identify as sexual and gender minority (SGM - lesbian, gay, bisexual, transgender, queer individuals) both represent minoritized groups of older adults, but little is known about the health experiences of older adults who are at the intersection of having dementia and also identify as SGM. This review explored the extent and nature of research focused on health and healthcare experiences of SGM people with dementia and/or their informal caregivers. A scoping review framework was used given the exploratory nature of the project’s purpose. Health librarians comprehensively searched four databases (MEDLINE, CINAHL, PsycINFO, AgeLine) to identify relevant studies. A total of 8,137 unique titles and abstracts were reviewed by 2 independent reviewers. Forty citations were determined relevant and reviewed by full text. Eight studies met inclusion criteria and were analyzed thematically. Study methods were quantitative cross-sectional (n=2), qualitative exploratory (n=4), and a mixed methods case study (n=1). Studies were published from 2010–2022. Sample sizes ranged from 1 to 415 (M=113, SD=116). Four studies focused on caregivers, 2 on persons with dementia, and 1 on dyads. Themes emerged pertaining to caregiver health and well-being, identity, relationships, disclosure, discrimination, and safety. Findings highlight the significance of inclusive care that addresses intersecting psychosocial and health-related identities (e.g., cognitive status, SGM status, race/ethnicity, substance abuse history, multimorbidity) and that protects the rights of families of choice. More research is needed to better understand how sociopolitical structure influences dynamics between cognitive health and SGM status among older adults.

